# Supplementary material for: A Broad-Spectrum Phage Endolysin (LysCP28) Able to Remove Biofilms and Inactivate Clostridium perfringens Strains
Source: Foods. 2023 Jan 15;12(2):411. doi: 10.3390/foods12020411 (PMC9858456; doi:10.3390/foods12020411)

Supplementary material

Table S1. Bacterial strains used in this work. *C. perfringens* reference strains and isolated from different sources were used to determine the lytic spectrum of endolysin LysCP28. Selected gram-positive and gram-negative bacteria were also included. The *C. perfringens* serotype and the origin of each strain were indicated. Source of each strain is included in the strain name: BG chicken liver, J chicken manure, Y goat, N cow. Place of isolation is (1) BinZhou city; (2) Rizhao city; (3) Xi'an City; (4) Mianyang City; (5) Sihong City.

Strain	Type	LysCP28	Source	Region
BG1	A	++	Chicken liver	(1)
BG3	A	++	Chicken liver	(1)
BG4	A	++	Chicken liver	(1)
BG5	A	++	Chicken liver	(1)
BP3	A	+	Chicken spleen	(1)
BX1	A	+	Chicken heart	(1)
BX6	A	-	Chicken heart	(1)
BP1	A	++	Chicken spleen	(1)
BP2	A	+	Chicken spleen	(1)
BP5	A	+	Chicken spleen	(1)
BX4	A	++	Chicken heart	(1)
BX5	A	-	Chicken liver	(1)

BC5	A	++	Chicken intestinal	(1)
BC4	A	++	Chicken intestinal	(1)
BC7	A	++	Chicken intestinal	(1)
BC8	A	++	Chicken intestinal	(1)
G1-12	A	++	Chicken intestinal	(1)
G2-2	A	-	Chicken intestinal	(1)
G2-4	A	-	Chicken intestinal	(1)
G3-2	A	-	Chicken intestinal	(1)
BG7	A	+	Chicken liver	(1)
BC3	A	+	Chicken intestinal	(1)
BX3	A	+	Chicken heart	(1)
BX9-1	A	+	Chicken heart	(1)
BG9-1	A	-	Chicken liver	(1)
RZ-1	A	+	Chicken intestinal	(2)
RZ-4	A	+	Chicken intestinal	(2)
RZ-5	A	++	Chicken intestinal	(2)
ZA3	A	++	Chicken intestinal	(2)
ZA1	A	++	Chicken intestinal	(2)
ZA4	A	+	Chicken intestinal	(2)
ZA2	A	++	Chicken intestinal	(2)
J2	A	++	Chicken manure	(3)
J2A	A	+	Chicken manure	(3)

J112	A	+	Chicken manure	(3)
J120	A	+	Chicken manure	(3)
J121	A	+	Chicken manure	(3)
J21	A	+	Chicken manure	(3)
J22	A	++	Chicken manure	(3)
J19	A	+	Chicken manure	(3)
J119	A	+	Chicken manure	(3)
J25	A	++	Chicken manure	(3)
J125	A	++	Chicken manure	(3)
J126	A	-	Chicken manure	(3)
J9	A	+	Chicken manure	(3)
J9A	A	+	Chicken manure	(3)
J11	A	+	Chicken manure	(3)
J111	A	+	Chicken manure	(3)
J15	A	+	Chicken manure	(3)
J115	A	+	Chicken manure	(3)
J10	A	+	Chicken manure	(3)
J12	A	+	Chicken manure	(3)
J20	A	+	Chicken manure	(3)
A1	A	-	Chicken manure	(3)
Z-6	A	-	Chicken manure	(3)
S-3	A	-	Chicken manure	(3)

D4	A	-	Chicken manure	(3)
7Y	A	+	Goat	(3)
70Y	A	+	Goat	(3)
75Y	A	-	Goat	(3)
3Y	A	+	Goat	(3)
40Y	A	++	Goat	(3)
36Y	A	-	Goat	(3)
76Y	A	+	Goat	(3)
53Y	A	+	Goat	(3)
21N	A	-	Cow	(3)
4N	A	++	Cow	(3)
12N	A	+	Cow	(3)
121N	A	++	Cow	(3)
131N	A	+	Cow	(3)
33N	A	-	Cow	(3)
106N	A	+	Cow	(3)
42N	A	+	Cow	(3)
4AN	A	++	Cow	(3)
31N	A	+	Cow	(3)
1N	A	+	Cow	(3)
1AN	A	+	Cow	(3)
111N	A	-	Cow	(3)

11N	A	+	Cow	(3)
5N	A	-	Cow	(3)
MY1-1	A	-	Chicken	(4)
MY1-2	A	+	Chicken	(4)
MY2-2-1	A	+	Chicken	(4)
MY2-3-1	A	-	Chicken	(4)
CP-T14	A	+	Milk	(5)
C. perfringens ATCC 13124	A	+	-	-
C. perfringens CMCC 64722	A	+	-	-
C. perfringens CMCC 64723	A	+	-	-
C. perfringens CMCC 64724	A	+	-	-
C. perfringens CVCC 54	B	++	-	-
C. perfringens CVCC 81	D	++	-	-
C. perfringens CVCC 1153	C	++	-	-
S. aureus ATCC 25923	-	-	-	-
S. Enteritidis ATCC 13076	-	-	-	-
E. coli ATCC 25922	-	-	-	-

Strong lytic zone (++), Lytic zone (+) or no lytic zone (-).

Figure S1

Figure S1. Sequence analysis and phylogenetic comparison of the endolysin LysCP28. A). Evolutionary distances computed using the Poisson correction method and expressed as amino acid substitutions per site. Constructed by maximum parsimony with 1000 replications. B) Functional prediction of catalytic domains in the LysCP28 sequence. The sequence function was predicted by Pfam and Conserved Domain Database. The domain hits: (1) GH25_Lytic-like: The Lytic lysozyme of Streptococcus pneumoniae is a bacterial cell wall hydrolase. (2) Glyco_hydro_25: Glycosyl hydrolases family 25. (3) Acm: Lyzozyme M1(1,4-beta-N-acetylmuramidase), GH25 family [Cell wall/membrane/envelope biogenesis] (4) SH3_3: Bacterial SH3 domain. (5) SH3b: Bacterial SH3 domain homologues.

A.

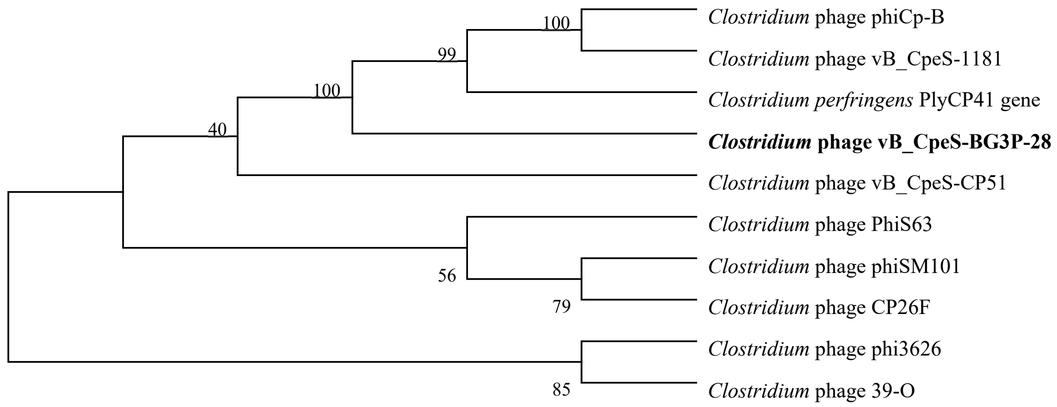

B.

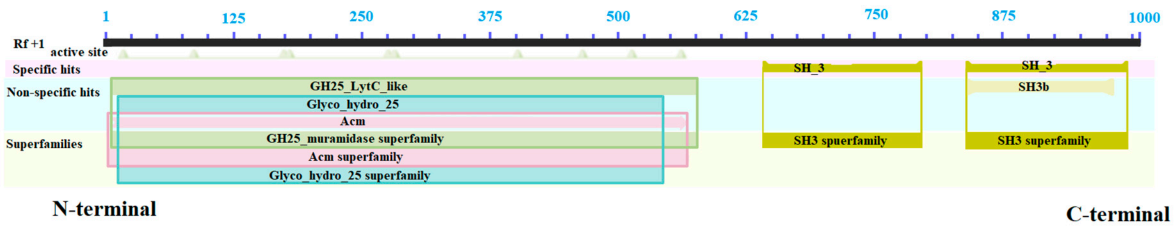

Figure S2

Figure S2. Overexpression and purification of *C. perfringens* endolysin LysCP28. Recombinant bacterial cultures were induced and protein was purified using the Ni-NTA columns. Each fraction was analyzed by SDS-PAGE: 1, Medium supernatant of recombinant bacteria; 2, Ultrasound supernatant of recombinant bacteria. Line 3, Flowthrough；lines 4-8：Fractions obtained after elution with Imidazole 25 mM, 50 mM, 100 mM, 200 mM, 250 mM. Line M: Standard molecular weight (please indicate commercial name or brand).

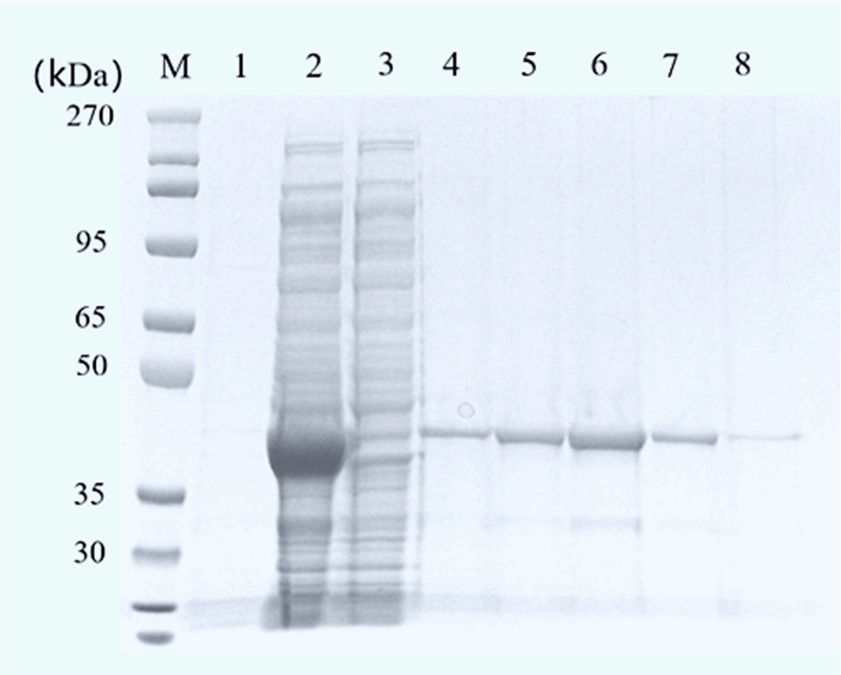

Supplement: Supplementary file 1 [file foods-12-00411-s001.zip › foods-2108077-Supplementary.pdf]
